# Clinical Experience of Laser Angioplasty for the Cardiovascular Disease

**DOI:** 10.1155/DTE.2.11

**Published:** 1995

**Authors:** Masayoshi Okada, Masato Yoshida, Yoshihiko Tsuji

**Affiliations:** Department of Surgery Division II Kobe University School of Medicine 7-5-2 Kusunoki-Cho, Chuo-ku Kobe 650 Japan

## Abstract

In recent years, lasers are being utilized in cardiovascular surgery. Since the 1980's we have investigated angioplasty using an Argon laser for patients with obstructive arterial diseases. This technique aims to open the obstructive arterial lumen. Based on the excellent results of experimental studies, the technique has been clinically applied. Laser angioplasty was carried out in 84 patients with stenotic or obstructive lesions occluding more than 75% of peripheral and coronary arteries angiographically. They consisted of 74 cases with intermittent claudication and 10 cases with angina pectoris. Laser angioplasty for the peripheral arterial disease was performed under local anesthesia in the inguinal region under angioscopic guidance. On the other hand, laser coronary angioplasty was simultaneously undertaken at the time of coronary artery bypass grafting for a patient with multiple coronary stenoses. The initial success rate by laser angioplasty for the peripheral artery was 91% in the stenotic lesions and 71% in the obstructive lesions. The cumulative patency rate was 94% in the stenotic lesions and 83% in the occlusive lesions. A follow-up study of 66 months was carried out for patients with clinical success, excluding the cases where an angiogram showed occlusion within 1 week after laser angioplasty. Consequently, excellent long-term results could be clinically obtained. Based on the satisfactory results in the peripheral artery, coronary laser angioplasty was employed in 10 patients with angina pectoris. There were no complications by laser. Thus, the feasibility of laser application was apparently confirmed and laser angioplasty might be recommended for patients with atherosclerotic changes, especially for small arteries.


*Diagnostic and Therapeutic Endoscopy*, 1995, Vol. 2, pp. 11–18
Reprints available directly from the publisher
Photocopying permitted by license only

© 1995 Harwood Academic Publishers GmbH
Printed in Singapore

### Clinical Experience of Laser Angioplasty for the Cardiovascular Disease

MASAYOSHI OKADA, MASATO YOSHIDA and YOSHIHIKO TSUJI

*Department of Surgery, Division II, Kobe University School of Medicine*

*(Received December 27, 1993; revised February 21, 1995)*

In recent years, lasers are being utilized in cardiovascular surgery. Since the 1980's we have investigated angioplasty using an Argon laser for patients with obstructive arterial diseases. This technique aims to open the obstructive arterial lumen. Based on the excellent results of experimental studies, the technique has been clinically applied. Laser angioplasty was carried out in 84 patients with stenotic or obstructive lesions occluding more than 75% of peripheral and coronary arteries angiographically. They consisted of 74 cases with intermittent claudication and 10 cases with angina pectoris. Laser angioplasty for the peripheral arterial disease was performed under local anesthesia in the inguinal region under angioscopic guidance. On the other hand, laser coronary angioplasty was simultaneously undertaken at the time of coronary artery bypass grafting for a patient with multiple coronary stenoses. The initial success rate by laser angioplasty for the peripheral artery was 91% in the stenotic lesions and 71% in the obstructive lesions. The cumulative patency rate was 94% in the stenotic lesions and 83% in the occlusive lesions. A follow-up study of 66 months was carried out for patients with clinical success, excluding the cases where an angiogram showed occlusion within 1 week after laser angioplasty. Consequently, excellent long-term results could be clinically obtained. Based on the satisfactory results in the peripheral artery, coronary laser angioplasty was employed in 10 patients with angina pectoris. There were no complications by laser. Thus, the feasibility of laser application was apparently confirmed and laser angioplasty might be recommended for patients with atherosclerotic changes, especially for small arteries.

**KEY WORDS:** atherosclerosis, intermittent claudication, angina pectoris, laser angioplasty, angioscopy

### INTRODUCTION

In recent years, endovascular interventions such as balloon angioplasty, atherectomy and the stenting method have been clinically applied for patients with atheromatous plaques of the peripheral- and the coronary arteries (1,3). Among them, restenosis after endovascular intervention has been obviously confirmed in 30–40% of the patients who underwent percutaneous transluminal coronary angioplasty (2). However, there are some problems in maintaining long-term patency by means of endovascular techniques such as balloon techniques and atherectomy. For these problems, we have experimentally investigated the effect of an Argon laser on vaporization of the atheromatous plaques. Based on our excellent experimental studies, a laser was utilized for patients with intermittent claudication, angina pectoris, and ischemic ulcer of the lower extremity. Subsequently, the feasibility of laser angioplasty could be disclosed in the field of cardiovascular surgery (4).

Address for correspondence: Masayoshi Okada, M. D., Department of Surgery, Division II, Kobe University School of Medicine, 7-5-2 Kusunoki-Cho, Chuo-ku, Kobe 650, Japan.

### MATERIALS AND METHODS

In cardiovascular surgery, Argon, Nd-YAG, Ho-YAG and Excimer lasers have been widely employed all over the world. An Argon laser (Trimedyne/Laser Ionics, Model 5567, and HGM, Endocoagulator Model 20S, USA) was utilized for the vaporizing of atherosclerotic plaques in this study (4). Animal care was in compliance with the "Principles of Laboratory Animal Care" and the "Guide for Care and Use of Laboratory Animals" (NIH Publication No. 80-23, revised 1985).

First of all, the relationship between laser energy and reactions of the aortic walls to the laser was experimentally evaluated using adult mongrel dogs. A metal tip probe (Laserprobe PLR-Flex or Laserprobe PLR-Plus, Trimedyne

Inc. CA, USA) was mainly used. Consequently, it could be confirmed that optimal conditions for laser angioplasty were 6W in output and 3 sec in irradiation time for each shot. On the other hand, a metal tip probe with thermal feedback control system (HGM, Endocoagulator, Model 20S, and Laserprobe, PLAC, HGM, Salt Lake City, UT USA) was applied, for which the adequate tip temperature for vaporizing the atheromatous plaques was 200°C and the irradiation time was 5 sec for each shot. These conditions were almost the same laser energy for adequacy, that is, in the case with 6W and 3 sec using the MTP, the intimal layer was vaporized, and with 8W and 3 sec, the crater reached the media. Then, with 8W and 3 sec using the BEP, arterial perforation occurred immediately. On the other hand, in the case with thermal feedback control system the arterial crater was deepened by an increase in the temperature of the MTP (Fig. 1). In the case with 100~150°C in the temperature of the MTP, the intimal surface was slightly vaporized, with 200~300°C, the crater reached deeper initimal layer. In the case with more than 300°C, the media was also vaporized. Consequently, it was considered that suitable temperature of the MTP was 200°C.

At the time of laser angioplasty, the use of an angioscope was inevitable for observing the inside of the arteries before and after laser irradiation (1). At present, laser angioplasty has been carried out for 84 patients with atherosclerotic changes of the peripheral and coronary arteries. This includes 70 men and 14 women, ranging in age from 36- to 81-years-old with an average of 65.7 ± 9.8. These patients consisted of 71 cases with intermittent claudication, 2 cases with rest pain and one case with a refractory ulcer in the leg, including 10 cases with angina pectoris.

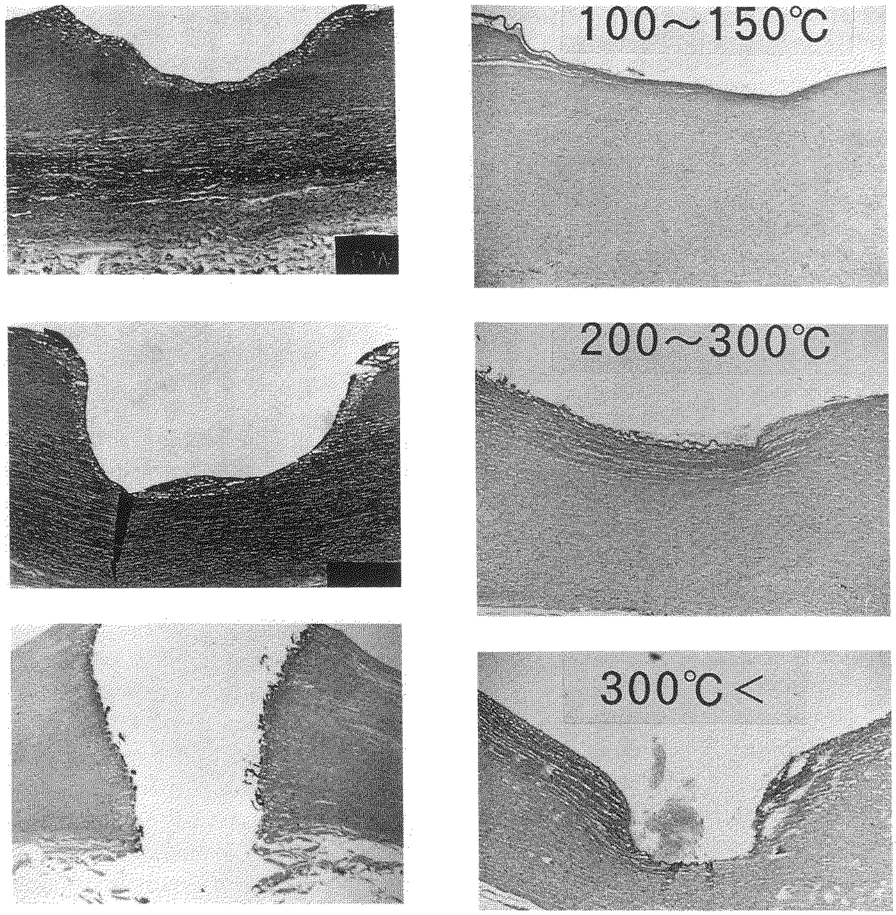

(a) (b)

**Figure 1** Laser energy and microscopic reactions of the arterial wall a) With metal tip probe b) With metal tip probe included thermal feedback control system.

Angiography for the peripheral and coronary arteries was undertaken in all patients and their operative indication was clearly decided by clinical symptoms and angiographic findings with severe stenosis of more than 75% of the internal diameter and total occlusion of the arteries (1). The length of occlusive and stenotic lesions in the peripheral arteries ranged from 0.5 cm to 45 cm with a mean of 7.6 ± 8.8 cm.

As the method of laser angioplasty, a sheath catheter was percutaneously inserted proximally for the iliac lesions and distally for the femoral and popliteal lesions under local anesthesia in the inguinal region. After this procedure, an angioscope (0.75 mm in diameter) was inserted through the sheath catheter and the surface of the atherosclerotic plaques in the artery was observed. At this time, it is very important to wash out the blood with saline for clear observation. Through this procedure, the inside of the artery could be precisely observed. There are several sizes of angioscope, such as 0.75 mm or 0.45 mm in diameter. From the standpoint of image clarification, an angioscope of 0.75 mm in diameter was the most useful instrument. Thereafter, laser irradiation was carefully initiated to make a laser hole for the occlusive lesion under angioscopic guidance using a bare-ended laser fiber and metal tip probe. In the case with a calcified occlusion at the site of the femoral artery, this artery was first exposed and a sheath catheter was directly inserted to the atheromatous plaques under direct vision. A guide-wire was then passed through a laser hole which was previously created using a bare-ended laser fiber. Then laser ablation was carefully performed using a metal tip probe (2.0–2.5 mm in diameter) inserted over the guide-wire. In this study, 6W in output and 3 sec in irradiation time, or 200°C and 5 sec using a metal tip probe with thermal feedback control system were utilized as optimal conditions for each laser ablation. Laser ablations were continued to eliminate atheromatous plaques under angioscopic and fluoroscopic guidance, until recanalization and a wide opening of the vessel lumen could be observed. Consequently, the conditions of 6W and 3 sec were applied for 23 patients. For the remaining 51 cases, the feedback control system was employed. Moving the metal tip probe slowly in the artery, it is very important to prevent vasoconstriction and fragmentation of the atheromatous plaques during laser ablation. Then, in cases where dilatation was inadequate by lasing, percutaneous transluminal angioplasty (PTA) was additionally undertaken. All clinical results were expressed as the mean standard deviation. The unpaired t-test was used to test for differences in variables between the two measurements, and a P-value less than 0.05 was accepted as statistically significant.

### RESULTS

### 1) Laser Angioplasty For Peripheral Arterial Diseases

At present, 74 patients (93 lesions) have been treated by laser angioplasty. This includes 61 men and 13 women who had intermittent claudication, rest pain, and refractory limb ulcers. They consisted of 73 patients with arteriosclerosis obliterans and only one with thromboangitis obliterans.

Forty-one among 93 lesions were in the iliac region, and 48 lesions existed in the femoro-popliteal region. The remaining 4 lesions were thrombogenic stenoses of implanted vascular grafts and the anastomotic site of the saphenous vein after coronary artery bypass grafting. Forty-four lesions revealed stenotic changes, and 49 lesions showed obstructive findings. The length of lesions ranged from 0.5 cm to 45.0 cm with a mean of 7.6 cm in length by angiography (Table 1). The criteria of clinical success were evaluated as follows: a) relief of symptom, b) improvement of arterial pulsation, c) increase of ankle pressure index of more than 0.2, and d) residual stenosis of less than 50% in the internal diameter of the lased arteries. Based on these examinations, all cases were strictly evaluated. Subsequently, a success rate of 91% could be found in the cases with stenotic lesions. On the contrary, the success rate was 71% in the cases with occlusive lesions (Table 2). Further, from the length of the lesion, a clinical success rate of 97% was observed even in the cases with femoro-popliteal lesions within 10cm in length (Fig. 2). However, in the cases with lesions of more than 10cm in length, the rate decreased to 75%.

On the other hand, a clinical success rate of 86% was obtained in the cases with iliac lesions of less than 5 cm in length. Thus, there were significant differences in comparison with the success rate and the length of the lesion

**Table 1** Patient Characteristics of Laser Angioplasty for Chronic Obstructive Arterial Diseases

| | | | |
|---|---|---|---|
| Case | 74 cases (87 limbs, 93 lesions) | | |
| Gender | Male 61 cases, Female 13 cases | | |
| Age | 43 ~ 82 Y (65.7 ± 9.8 Y) | | |
| Disease | ASO 73 cases, TAO 1 case | | |
| Chief | Intermittent claudication | 71 | cases |
| Complaint | Rest pain | 2 | cases |
| | Refractory ulcer | 1 | case |
| Location | Iliac | 41 | lesions |
| | Fem-pop. | 48 | lesions |
| | Prosthetic graft stenosis | 1 | lesion |
| | Graft stenosis | 1 | lesion |
| | SVG graft stenosis | 2 | lesions |
| Severity | Occlusions | 49 | lesions |
| | Stenosis | 44 | lesions |
| Length | 0.5 ~ 45 cm (7.6 ± 8.8 cm) | | |

(Kobe Univ. Surg II: 93.6)

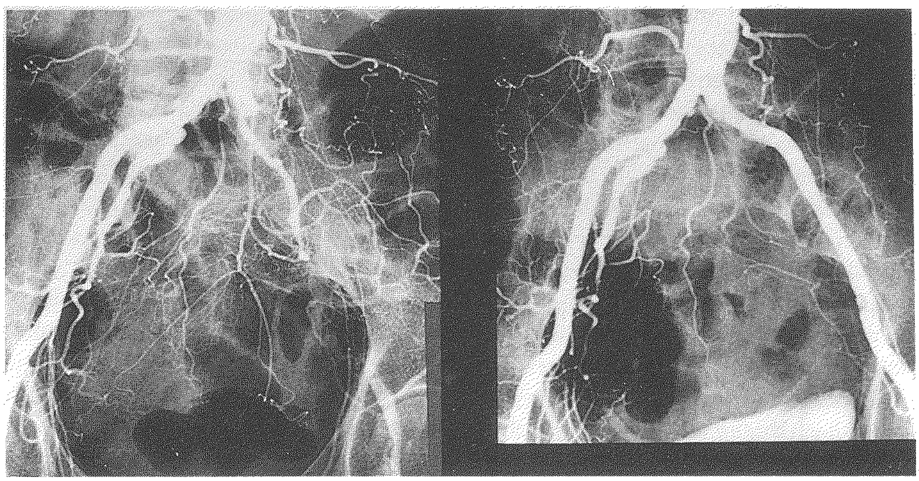

a)

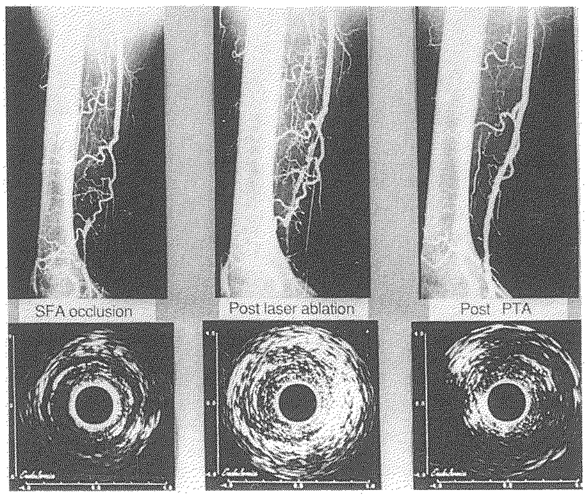

b) (1) (2) (3)

**Figure 2** Percutaneous laser angioplasty for the peripheral artery. a) Laser angioplasty for the occlusion of the left common iliac artery. b) Laser angioplasty for the occlusion of the right superficial femoral artery under intravascular ultrasound (1) before lasing (2) after lasing (3) after PTA.

between both groups (Table 3). The tortuorsity and severe calcification in these regions were considered to be the reason for this significance. The percentage of stenosis of the arterial lesions improved from 91.5 ± 11.0% before, to 12.9 ± 18.8% after, and the ankle pressure index (API) increased from 0.49 ± 0.19% before, to 0.91 ± 0.16% after, in the cases with clinical success.

A long-term follow-up study of 66 months was carried out on 63 limbs with clinical success by angiography, measurement of ankle pressure index and clinical symptoms except for angioscopy. Consequently, the cumulative patency rate in the follow-up of 66 months was 94% for the stenotic lesions and 83% for the occlusive lesions (Fig. 3).

**Table 2** Clinical Success Rate After Laser Angioplasty

*(I) Stenotic lesion*

| | *Mean length (cm)* | *No of lesions* | *Clinical success* |
|---|---|---|---|
| Femoro-popliteal lesion | 6.4 ± 8.5 | 22 | 20 (91%) |
| Iliac lesion | 2.0 ± 1.4 | 22 | 20 (91%) |
| Total | 4.2 ± 6.4 | 44 | 40 (91%) |

*(II) Occlusive lesion*

| | *Mean length (cm)* | *No of lesions* | *Clinical success* |
|---|---|---|---|
| Femoro-popliteal lesion | 12.6 ± 11.4 | 29 | 25 (86%)]* |
| Iliac lesion | 7.7 ± 5.1 | 20 | 10 (50%)]* |
| Total | 10.6 ± 9.6 | 49 | 35 (71%) |

* P < 0.05

**Table 3** Clinical Success Rate in the Relationship between the Location and the Length of the Stenotic Changes

*(1) Femoro-popliteal lesion*

| *Length* | *Mean length (cm)* | *No. of lesions* | *Clinical success* |
|---|---|---|---|
| < 10cm | 3.3 ± 2.4 | 33 | 32 (97%)]* |
| > 10cm | 22.0 ± 9.1 | 18 | 13 (72%)]* |
| Total | 9.9 ±10.6 | 51 | 45 (88%) |

* p < 0.01

*(II) Iliac lesion*

| *Length* | *Mean length (cm)* | *No. of lesions* | *Clinical success* |
|---|---|---|---|
| < 5cm | 2.1 ± 1.0 | 28 | 24 (86%)]* |
| > 5cm | 10.1 ± 4.4 | 14 | 6 (43%)]* |
| Total | 4.7 ± 4.6 | 42 | 30 (71%) |

* p < 0.01

### 2) Laser Angioplasty For Coronary Arterial Diseases

Laser coronary angioplasty was intraoperatively carried out for 10 patients with multiple stenoses in a coronary artery (Table 4).

This included 9 men and one woman, ranging in age from 39 to 67 years old with a mean of 58. A metal tip probe (1.5–2.0 mm in diameter) was used with angioscopic guidance, by which laser angioplasty could be safely done in all cases (Fig. 4). First of all, a coronary incision was made longitudinaly and the angioscope was inserted prior to the stenotic lesion. Then the characteristics of the atheronamous plaque were observed. Thereafter, the metal tip probe was passed to the lesion to vaporize the plaques under chilled heart protection. In the case of laser coronary angioplasty, the optimal conditions were 4–5 W in output and 2 sec in irradiation time for each shot. In addition to this, the diameter ratio between the metal tip probe and the coronary artery

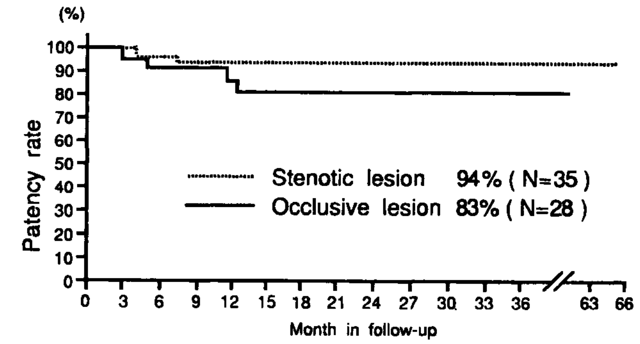

**Figure 3** Cumulative patency rate after laser angioplasty for the peripheral artery was an important factor for the prevention of coronary vasospasm during laser irradiation. That is, a smaller size metal tip probe, less than 30% of the diameter of the coronary artery, should be employed. The patients are doing well without severe complications throughout the treatment.

Consequently, laser coronary angioplasty was a useful procedure for multiple stenoses in a coronary artery, saving time in the operation and maintaining the long-term patency rate of coronary artery bypass grafting, because of mininized invasiveness.

### DISCUSSION

In recent years, Argon, Nd-YAG, Ho-YAG, and Excimer lasers have been widely utilized for laser angioplasty (11,20). The optimal conditions for laser angioplasty by Argon laser were 6W in output and 3 sec in irradiation time, or 200 in output and 5 sec in irradiation time in our serial experimental studies (17,18).

On the other hand, several laser conditions (6–12W in output, 3–10 sec in irradiation time) have been reported according to the kind of laser system and laser fiber. At the time of laser angioplasty, angioscopic and intravascular ultrasound guidance were necessary to evaluate the characteristics of the atheromatous plaques and the arterial wall before and after the laser ablation. And sometimes, it was angioscopically recognized that the proximal-and the distal portions were composed of tight atheromatous plaques and the middle portion was not occluded or filled with fresh thrombus.

Based on our clinical experience, laser angioplasty should be recommended to eliminate, or reduce the stenotic or occlusive changes of short segments within 10cm in length for a peripheral artery, and within 2cm in length for a coronary artery.

**Table 4** Clinical Experience of Laser Angioplasty for the Coronary Heart Disease

| *Case* | *Age* | *Sex* | *Symptom* | *CAG* | | | *Bypass* | *Site of Laser OTCA and its conditions* |
|---|---|---|---|---|---|---|---|---|
| 1) K.N. | 59 | M | UAP | LMT | (5) | 90% | LAD | |
| | | | | LAD | (7) | 75% | D1 | LAD |
| | | | | | (9) | 90% | RCA | (6 w, 3 sec, 3 times) |
| | | | | RCA | (3) | 50% | | |
| 2) I.K. | 41 | M | PIA | LAD | (6) | 90% | | |
| | | | | | (7) | 75% | LAD | LAD |
| | | | | Cx | (11) | 99% | | (6 w, 2 sec, 3 times) |
| | | | | RCA | (1) | 100% | | |
| 3) S.K. | 61 | F | PIA | LAD | (6) | 90% | | |
| | | | | | (9) | 90% | LAD | RCA |
| | | | | Cx | (13) | 100% | RCA | (6 w, 3 sec, 7 times) |
| | | | | RCA | (1) | 100% | | |
| 4) T.Y. | 56 | M | UAP | LAD | (6) | 75% | LAD | |
| | | | | Cx | (13) | 100% | Cx | RCA |
| | | | | RCA | (2) | 90% | RCA | (6 w, 3 sec, 5 times) |
| | | | | | (3) | 75% | | |
| 5) T.Y. | 39 | M | PIA | LAD | (7,8) | 75% | LAD | LAD |
| | | | | Cx | (13) | 90% | Cx | (6 w, 3 sec, 3 times) |
| | | | | RCA | (2) | 90% | RCA | |
| 6) M.N. | 67 | M | UAP | LAD | (6) | 100% | LAD | RCA |
| | | | | Cx | (14) | 90% | Cx | (5 w, 2 sec, 2 times) |
| | | | | RCA | (1) | 75% | RCA | 6 w, 3 sec, 3 times) |
| 7) T.A. | 65 | M | PIA | LAD | (6) | 100% | LAD | LAD |
| | | | | Cx | (13) | 99% | Cx | (6 w, 3 sec, 3 times) |
| | | | | RCA | (1) | 100% | RCA | |
| 8) M.M. | 65 | M | UAP | LAD | (6) | 100% | LAD | |
| | | | | Cx | (11) | 75% | Cx | LAD |
| | | | | | (12) | 90% | RCA | (4 w, 2 sec, 4 times) |
| | | | | RCA | (3) | 100% | | |
| 9) Y.N. | 61 | M | OMI | LAD | (6) | 100% | LAD | RCA |
| | | | | RCA | (2) | 90% | RCA | (2 w, 2 sec, 10 times) |
| 10) A.U. | 65 | M | UAP | LMT | (5) | 75% | LAD | LAD |
| | | | | Cx | (14) | 90% | Cx | (200 °C, 5 sec, 12 times) |
| | | | | RCA | (2) | 75% | RCA | |

PIA Postinfarction angina
AP Angina pectoris
UAP Unstable angina pectoris

Recently, laser angioplasty has been widely employed in the United States and European countries. The initial success rate by laser angioplasty has been reported to be 65–95% in comparison with 70–85% for percutaneous transluminal angioplasty. In our clinical series, the clinical success rate was 91% in the case of stenotic lesions in comparison with 71% for occlusive lesions.

Thus, satisfactory results by laser angioplasty were surely obtained (11,17).

On the other hand, the clinical success rate was 97% in cases with femoro-popliteal lesions of less than 10cm in length. Thus, there were slight differences in the laser effects between stenotic and occlusive lesions as well as in the relationship between initial success and clinical success in the long-term period. Cumberland has reported that 50 of 56 totally occluded arteries were recanalized by laser and that the ankle pressure index increased from 0.53 before to 0.84 after laser irradiation (12). In addition to this, technical success was 88% and clinical success was 77%. In Japan, similar results have been reported in recent years (19).

In general, complications in laser angioplasty include hematomas in the inguinal region, the subintimal pathway of the laser fiber, and dissective change of the artery by additional PTA. We have also experienced 6 dissections and 2 subintimal pathways of the arteries by additional PTA, and intimal injuries of laser probe and guide-wire. However, there was no urgent surgical intervention for them.

Thus, there are some differences in the clinical success due to indication, techniques and antithrombogenic management after laser angioplasty in several hospitals. On the other hand, intraoperative coronary laser angioplasty was a safe and effective procedure under direct vision (20,21).

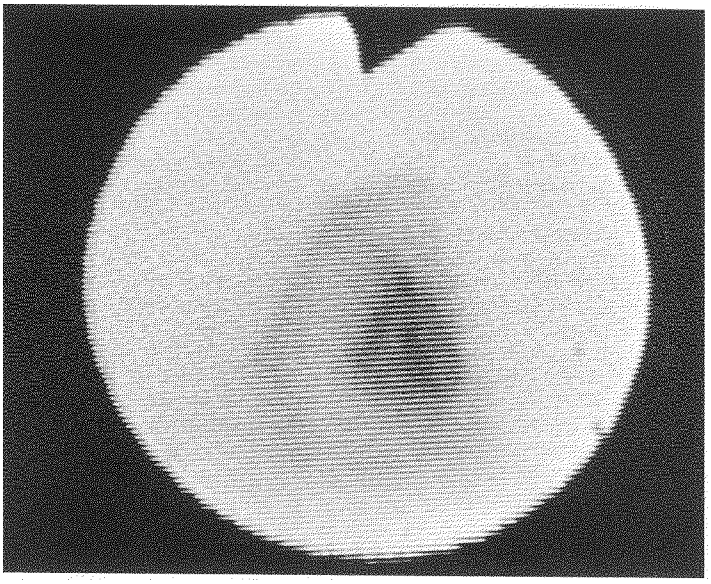

However, Litvack and Sanborn have reported a high incidence of complications after percutaneous transluminal a)

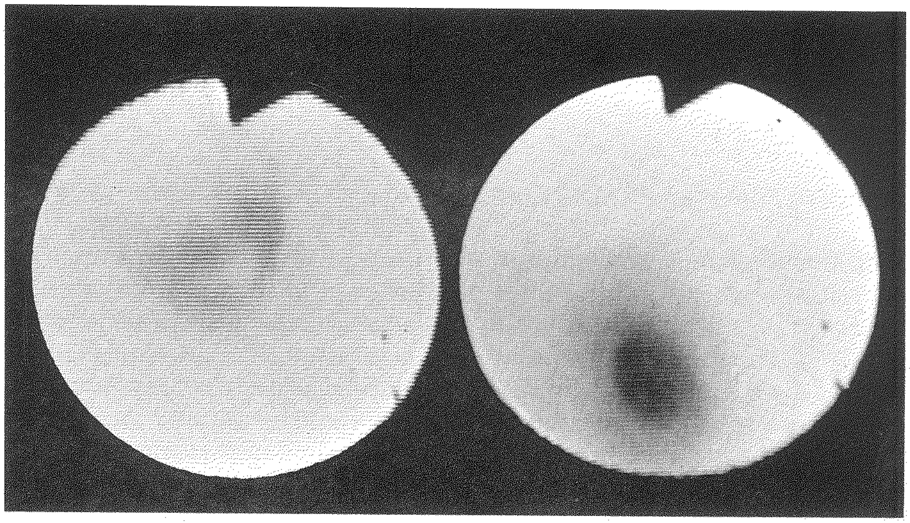

b)

**Figure 4** Laser coronary angioplasty. a) Endoscopic finding of severe stenosis of the coronary artery. b) Endoscopic findings before and after laser ablation.

laser coronary angioplasty (22,23). All cases treated by laser methods should be followed-up even in the long-term period.

In conclusion, laser angioplasty was effective to significantly open the lumen of occlusive and stenotic lesions of the peripheral artery. At present, excellent results have been revealed within 10cm in length for femoro-popliteal lesions. On the other hand, satisfactory results were obtained within 5 cm in length for iliac lesions. In the case of coronary arteries are good indications stenotic lesions within 2 cm in length. However, the indication for laser angioplasty will change in the long-term period.

### REFERENCES

1. Okada M, Yoshida M, Tsuji Y, Nakamura K. Laser angioplasty with vascular endoscope in the fields of the peripheral and the coronary artery. Surg & Med Lasers, 1988;1:53–60.
2. Vlestra RE. Percutaneous transluminal coronary angioplasty. Initial clinic experience. Mayo Clin Proc 1981;56:287–291.
3. Grüntzig A: Results from coronary angioplasty and implications for the future. Am Heart J 1982;103:779–783.
4. Gerrity RG, Loop FD, Golding LA, Ehrhart LA, Argenji ZB. Arterial response to laser operation for removal of atherosclerotic plaques. J Thorac Cardiovasc Surg 1983;85:408–421.
5. Sanborn TA, Faxon DP, Haudenshild CC, Ryan TJ. Experimental angioplasty: Circumferential distribution of laser thermal injury with a laser probe. J Am Coll Cardiol 1985;5:934–938.
6. Forrester JS, Litvack F, Grundfest WS. Laser angioplasty and cardiovascular disease. Am J Cardiol 1986;57:990–992.
7. Okada M, Tsuji Y, Yoshida M, Nakamura K. Clinical and experimental studies on laser application for coronary and the peripheral arterial disease. Revue Europ Technol Biomed 1990;12:27–31.
8. Abela GS, Norman S, Chen D, Feldman RL, Geaser EA, Conti CR. Effects of carbon dioxide, ND-YAG and Argon laser radiation of coronary atheromatous plaques. Am J Cardiol 1982;50:1199–1205.
9. Ginsburg R, Kim DS, Guthaner D, Mitchell RS. Salvage of an ischemic limb by laser angioplasty: Description of a new technique. Clin Cardiol 1984;7:54–58.
10. Diethrich EB, Timbadia E, Bahadir I, Colum K, Lenzen S. Argon laser assisted peripheral angioplasty. Vasc Surg 1988;22:77–87.
11. Ginsberg R, Wexler L, Mitchell RS. Percutaneous transluminal laser angioplasty for treatment of peripheral vascular disease. Radiology 1985;156:619–623.
12. Cumberland DC, Sanborn TA, Taylar DI, Moore DJ, Welsh CL, Greenfield AJ, Gulber JK, Ryan TJ. Initial clinical results with a laser probe in total periphral artery occlusions. Lancet 1986; 1:1457–1459.
13. Sanborn TA, Cumberland DC, Greenfield AJ. Percutaneous laser thermal angioplasty: Initial results and 1 year follow-up in 129 femoropoplitcal lesions. Radiology 1988;168:121–124.
14. Geschwind HJ, Tesseive B, Boussignac G. Percutaneous transluminal laser angioplasty in man. Lancet 1984;7:377–378.
15. Choy DS, Stertzer SH, Myler RK, Marco J, Fournial G. Human coronary laser recanalization. Clin Cardiol 1984;7:377–381.
16. Barbeau GR, Abela GS, Seequr JM, Friedl SE, Tomaru T, Giacomono PP. Temperature monitoring during peripheral thermo-optical laser recanalization in humans. Clin Cardiol 1990;13:690–695.
17. Okada M, Yoshida M, Tsuji Y, Nakamura K. Laser angioplasty for the peripheral and the coronary artery disease. Angéiologie 1990;42:121–127.
18. Uchida Y, Fujimori Y, Tomaru T, Kato A. Percutaneous angioplasty of chronic obstruction of peripheral arteries by a temperature controlled Nd-YAG laser system. J Intervent Cardiol 1992; 5:301–308.
19. Takekawa SD, Takahashi M, Kubo I, Tanaka J, Komas S. Laser angioplasty: our clinical results. Proc 3rd International Nd-YAG Symposium, 1986;pp333–337.
20. Lee G, Garcia JM, Chan MC, Corso PJ. Clinically successful longterm coronary recanalization. Am Heart J 1986;112:1323–1325.
21. Diethrich EB, Hanafy HM, Santiago OJ. Intraoperative coronary excimer laser angioplasty: Preliminary clinical experience. Angiology 1990;41:777–779.
22. Litvack F, Grund fest W, Hickey A, Jakubowski A. Percutaneous excimer laser coronary angioplasty: Results of the first 110 procedures. J Am Coll Cardiol 1990;15:25A.
23. Sanborn TA, Faxon DP, Kellett MA, Ryan TJ. Percutaneous coronary excimer laser assisted balloon angioplasty: Initial multicenter experience. J Am Coll Cardiol 1990;12:25A.

